# How we study cryptic species and their biological implications: A case study from marine shelled gastropods

**DOI:** 10.1002/ece3.10360

**Published:** 2023-09-05

**Authors:** Caren P. Shin, Warren D. Allmon

**Affiliations:** ^1^ Department of Earth and Atmospheric Sciences Cornell University Ithaca New York USA; ^2^ Paleontological Research Institution Ithaca New York USA

**Keywords:** cryptic species, evolution, Gastropoda, speciation, species delimitation, taxonomy

## Abstract

Methodological and biological considerations are intertwined when studying cryptic species. A potentially large component of modern biodiversity, the frequency of cryptic species among taxonomic groups is not well documented. The term “cryptic species” is imprecisely used in scientific literature, causing ambiguity when interpreting their evolutionary and ecological significance. This study reviews how cryptic species have been defined, discussing implications for taxonomy and biology, and explores these implications with a case study based on recently published literature on extant shelled marine gastropods. Reviewed gastropods were recorded by species. Records of cryptic gastropods were presented by authors with variable levels of confidence but were difficult to disentangle from inherent biases in the study effort. These complexities notwithstanding, most gastropod species discussed were *not* cryptic. To the degree that this review's sample represents extinct taxa, the results suggest that a high proportion of shelled marine gastropod species are identifiable for study in the fossil record. Much additional work is needed to provide a more adequate understanding of the relative frequency of cryptic species in shelled marine gastropods, which should start with more explicit definitions and targeted case studies.

## Graphical abstract

Methodological and biological considerations are inseparable when studying cryptic species. Using shelled marine gastropods, we review how cryptic species have been defined, reported, and the potential implications of cryptic species occurrences for taxonomy and evolution. We find most gastropod species are considered not cryptic, suggesting many species can be confidently identified and studied for living and fossil taxa.

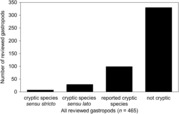

## 1 INTRODUCTION

Cryptic species, frequently defined as species that are morphologically difficult to diagnose, pose both theoretical and practical challenges to study. The term is frequently used ambiguously (Struck et al., 2017), and interchangeably with other phrases (e.g., “sibling species,” “species complexes”), making it difficult to draw ecological and evolutionary conclusions (especially at a macroevolutionary level; Chenuil et al., 2019; Fišer et al., 2018; Struck et al., 2017). Investigating cryptic species necessitates clear concepts of species and their delimitation, which affect how cryptic species are reported and discussed. The interconnectedness between methodology and potential for biological insight underlies work on cryptic species. Are cryptic species the result of insufficient study and limitations of our current methods (e.g., “The eventual elimination of all such cases may be considered the most tangible result of taxonomic work,” Mayr, 1940; see also, Bateman, 2022; Korshunova et al., 2019; Martynov & Korshunova, 2022; Mayr, 1942, 1963; Mayr & Ashlock, 1991, p. 91–93; Monro, 2022)? Or are cryptic species real biological entities that can inform us about processes such as speciation by different mechanisms or timescales (from thousands of years, e.g., red alga, Payo et al., 2013; reef fish, Hench et al., 2022; to millions of years, e.g., scyphozoan cnidarians, Dawson & Jacobs, 2001; amphipods, Fišer et al., 2018; annelids, Cerca et al., 2019)?

Cryptic species have been reported among many biological groups and are frequently supposed to be a large part of extant biodiversity (e.g., Bickford et al., 2007; Mayr, 1963; Pfenninger & Schwenk, 2007). While cryptic species reporting has accelerated dramatically with increased accessibility of genetic sequencing, this may not reflect an accurate estimate of how common cryptic species actually are, especially if different taxonomic groups may each have variable proportions of cryptic taxa (Pérez‐Ponce de León & Poulin, 2016), and there are methodological concerns with the chosen method (e.g., for DNA “barcoding,” as reviewed by DeSalle & Goldstein, 2019; Frézal & Leblois, 2008; Taylor & Harris, 2012), and sampling (e.g., uneven study effort among groups, Tronteij & Fišer, 2009; geographical extent sampled, Bergsten et al., 2012; insufficient number of specimens, Meyer & Paulay, 2005; Phillips et al., 2019). While there are studies at lower taxonomic levels (within a species, e.g., Hebert et al., 2004; among related species, e.g., Shaw, 2000; comparisons among related genera, e.g., Chaban, Ekimova, Schepetov, Kohnert, et al., 2019), there are few rigorous reviews of specific groups (e.g., decapod crustaceans, Knowlton, 1986; black flies, Adler et al., 2010; parasitic worms, Poulin, 2011; helminths, Poulin & Pérez‐Ponce de León, 2018; polychaetes, Nygren, 2013; nematodes, Palomares‐Ruis et al., 2014; bryophytes, Renner, 2020; insects, Li & Wiens, 2022), or environments (e.g., marine setting, Knowlton, 1993, 2000), which may have specific conclusions that are applicable more widely (e.g., across phyla).

For marine phyla, estimates of cryptic species by specialists are highly variable and taxondependent (e.g., microbes, Pedrós‐Alió, 2006; eukaryotes, Leray & Knowlton, 2016; all phyla, Appeltans et al., 2012), ranging from “ubiquitous” (Knowlton, 1993), “tens of thousands” or “11%–43%” of accepted described species (based on expert opinion, Appeltans et al., 2012), to “4.41 cryptic species per nominal species” (based on literature, Chenuil et al., 2019). Unfortunately, most marine species are not as well‐known as some other groups (e.g., North American birds, Kerr et al., 2007; West Mediterranean butterflies, Vodă et al., 2015) due to operational limitations (e.g., marine habitats may be less accessible, many species are primarily known from preserved material) leading to the sentiment, “we simply do not know our squids, starfish and shrimp as well as ornithologists know their birds” (Knowlton, 2000, p. 83; also Knowlton, 1993). Marine cryptic species may occur more often in some higher taxa than others (estimates range from zero to “no basis to make an estimation” depending on taxa, Appeltans et al., 2012; Chenuil et al., 2019). Differences in cryptic species estimates may come from both methodology (e.g., uneven study effort, Pérez‐Ponce de León & Poulin, 2016, or distinct review techniques), and biology, for example, if groups with higher estimates are due to few externally visible characteristics (chemical, audio, or other nonvisible diagnostic features, which may be more difficult to study, Appeltans et al., 2012; Bickford et al., 2007; Knowlton, 1993).

Our poor knowledge of the relative frequency of cryptic species is a significant obstacle to progress in many areas of evolutionary biology, including the study of modern biodiversity and the functioning of communities, as well as the recognition of species in the fossil record. We cannot fully understand what we cannot accurately quantify (e.g., Allmon, 2016 and references therein). In this paper, we attempt to clarify the use of the "cryptic species" term, review potential implications of cryptic species, and estimate the frequency of cryptic species for one major set of taxa: extant shelled marine gastropods. Gastropods are abundant and diverse today as the largest constituent of mollusk diversity (73%–78% of named species, Ponder & Lindberg, 2020, or 50,000–55,000 species, MolluscaBase, 2023), with an extensive fossil record since the early Paleozoic (~540 million years ago). With 32,000–40,000 described living species, it is estimated only 23%–32% of marine gastropod diversity is known, with a growing discovery rate (Appeltans et al., 2012). We focus on marine gastropods that have shells as adults, a grouping that includes examples from all major clades: Caenogastropoda, Heterobranchia, Neritimorpha, Vetigastropoda, Neomphalina, and Patellogastropoda (Uribe et al., 2019), but notably excludes the Nudibranchia (sea slugs), which lose their shells in adulthood. Most gastropod species are first described using macroscopic shell characters (e.g., Bieler, 1992), and these conchological features can be well preserved and studied as fossils (e.g., Allmon & Smith, 2011; Ponder & Lindberg, 2020). Fossil gastropods have been the subject of influential studies of origination and extinction at the species level, and on the relationship between form, development, and evolution (see Allmon & Smith, 2011, and references therein). By selecting shelled marine gastropods, this review aims to bring together findings from species‐level studies of extant taxa to form conclusions, which may be applied to their fossil record.

## 2 HOW CRYPTIC SPECIES ARE STUDIED

### 2.1 Cryptic species term use

“Cryptic species” is a general label today for any species found to morphologically differ slightly or not at all from other known species, but there is a long history of widespread confusion in its use (Struck et al., 2017; Table 1). Separate biological “kinds” with few outward differences were noted as early as 1718 in birds (Winker, 2005, cited by Struck et al., 2017). This meaning is captured in German with the terms “double or dual species” or “sibling species” (“doppelarten” or “geschwisterarten,” as cited in Mayr, 1942, 1963, who references a history of these terms in Ramme, 1951; “geschwisterarten” may have been first published in Müller, 1874), or in French with “twin species” (“espèce jumelle,” as cited in Mayr, 1942, 1963; perhaps first published in Feer, 1890; still used today, Triplet, 2021). The English term “cryptic species” in this sense was probably first used by Darlington (1940), in reference to species that do not change their appearance despite genetic isolation due to mate choice. The synonymous term “sibling species” was translated into English from the earlier German and French terms by Mayr (1940) and was initially defined as multiple related species that are often considered or mistaken as one species because they are indistinguishable from each other. Since then, the terms “cryptic species” (usually referring to a species with no or little external diagnostic features, which makes them difficult to identify) and “sibling species” (two or more related species that are morphologically difficult to differentiate) have been used mostly interchangeably. Additional terms have been used, such as “pseudocryptic species” (formerly morphologically unidentifiable species that are found to have some unique characters) and associated phrases, including “cryptic speciation” (processes resulting in cryptic species) and “cryptic diversity” (the presence of many cryptic species in a study group). It remains unclear if putative “cryptic” or “sibling” species are different from typical species, and if so, what evolutionary processes produce them (e.g., Korshunova et al., 2019; Monro & Mayo, 2022; Sáez & Lozano, 2005). Several recent discussions have tried to untangle these issues (Chenuil et al., 2019; Struck et al., 2017).

**TABLE 1:** Definitions of common terms used to describe cryptic species and associated phrases, with notes on their use.

Term	Definition
Species descriptors	
Cryptic species (Darlington, 1940)	A species that does not change their appearance despite genetic isolation due to mate choice. Equivalent to Mayr (1940)
Cryptic species (most modern use)	A species which is morphologically (usually external characteristics) difficult to identify Often undefined in use and synonymous with sibling species (e.g., Lincoln et al., 1998; see below). Rarely referred to as aphanic species (e.g., Bolow, 2017). Recognizing formerly cryptic species is sometimes referred to as ‘decrypting’ species
Cryptic species (Struck et al., 2017)	A reproductively isolated species which does not have diagnostic morphology, and when compared to a species it is morphologically similar to, is less morphologically variable (implying stasis as an evolutionary cause for crypticity)
Cryptic species sensu stricto; cryptic species sensu lato (Chenuil et al., 2019; this study)	Cryptic species sensu stricto (‘in the strict sense’) are genetically distinct species which are confirmed to not have diagnostic morphology (at present with the data available; morphology may include external features, such as the shell, or internal anatomy, such as the radula). In this study, these are also species which were newly named Cryptic species sensu lato (‘in the broad sense’) are genetically distinct species that are difficult to visually identify but have some unique phenotypic character(s). In this study, these are also species which were formally described Both cryptic species sensu stricto and sensu lato assume morphological distinction indicates genetic differentiation “Cryptic genetically isolated units” (Chenuil et al., 2019) refer to both cryptic species sensu stricto and sensu lato, and may appear reproductively isolated but have the potential to interbreed if the taxon's range changes or a geographic barrier disappears; this term is not used in this review
Reported cryptic species (this study)	Taxa reported to be genetically distinguishable from others, but without sufficient other evidence to confirm a cryptic species status. Depending on the data available, reported cryptic species can be further categorized: those that are probably not cryptic (based on some morphological indication), putatively cryptic (based on genetic analyses), and unconfirmed (due to data deficiency)
Pseudo‐sibling species, or pseudo‐cryptic species	Species that were formerly morphologically undiagnosable, that later were found to have some differentiating features (e.g., Jörger & Schrödl, 2013). Some authors argue that this subdivision of cryptic species adds confusion (e.g., Korshunova et al., 2019)
Sibling species (modern use)	Translated into English from German (“doppelarten”, double or dual species; “geschwisterarten”, sibling species) and French (“espèce jumelle”, twin species) by Mayr (1940). Defined as two or more related species that are difficult to identify, whether using morphological or other non‐morphological characters (e.g., pheromones, behavior; Mayr, 1963) Often undefined and synonymous with cryptic species (modern use, e.g., Barrows, 2011; Hine, 2019; Lincoln et al., 1998). A Google Ngram search (Michel et al., 2010) indicates that “sibling species” was more frequently used than “cryptic species” until about 2020
Sister species	Two separate species which are each others' closest relatives. However, used in a similar manner or confused with species pair and sibling species
Species pair	Two separate species which have similar traits and overlapping geographic distributions (sympatric, as defined in Hine, 2019). Often used similarly or confused with sister species and sibling species
Species complex	A group of closely related species which are often, but not always difficult to identify (e.g., to describe disease vectors, Lane, 1997; mosquitoes, Harbach, 2012). Sometimes used with species group (an informal taxonomic grouping of species, e.g., Lincoln et al., 1998; Thain & Hickman, 2004) or species aggregate (e.g., as defined in Lincoln et al., 1998) Published uses date from the late 19th century (e.g., flowering plants, Brendel, 1887; Fernald, 1898; beetles, Casey, 1891; frogs, Hillis, 1988), and continues to be used widely
Associated phrases	
Crypsis or cryptic	Typically refers to camouflage or behavior(s) related to hiding (e.g., cryptic habitat, cryptic coloration, cryptic mate choice), or unexpected discoveries (e.g., cryptic introduction or cryptic invasion, cryptic genetic variation). ‘Morphological crypsis’ would describe taxa that are difficult to discriminate using morphology
Cryptic diversity	To allude to more than one cryptic species present in the studied taxon or larger group
Hyper‐cryptic	To describe many occurrences of cryptic species in a study group (e.g., Adams et al., 2014)
Cryptic speciation	Probably first cited in the 1950s but not defined (Price, 1958), this indicates evolutionary process(es) leading to the formation of cryptic species (e.g., in gastropods, Fernandes et al., 2021; Sanjuan et al., 1997). It is unclear if this is in reference to one specific, or several different pathways

### 2.2 Taxonomic treatment of cryptic species

The significance of cryptic species is hard to assess in part because there is no consistent approach to dealing with them taxonomically after they are discovered. Cryptic species are typically not included, integrated, or formally described in subsequent work (Fišer et al., 2018; Struck et al., 2017), which can lead to an underestimation of biodiversity (e.g., regional records, Witman et al., 2004; global estimates, Mora et al., 2011), as well as potential misidentification of species‐specific interactions, or incorrect evaluation of species for conservation (Bickford et al., 2007; Bolow, 2017; Chenuil et al., 2019; Struck et al., 2017).

Some authors have recommended formal description of cryptic taxa to allow increased inclusion in subsequent studies (e.g., Fišer et al., 2018; Pante, Schoelinck, & Puillandre, 2015; Puillandre, Cruaud, & Kantor, 2010), and addition to slow‐growing taxonomic knowledge (“the Linnean shortfall,” which is the gap between the number of species in nature and those described, e.g., Walters et al., 2021). However, formally describing cryptic species after their initial detection may be uncommon due to concerns about sufficient sampling, data considered, or because species description falls outside the scope of a nontaxonomic study. Taxonomic follow‐up may require significant effort (e.g., more specimens) and expertise (such as taxonomists, which may be lacking, sometimes referred to as “the taxonomic impediment,” e.g., Engel et al., 2021), so even if this is conducted, it may be many years between when a taxon is first recognized and a formal species description (e.g., decades for angiosperms, Goodwin et al., 2020). Additionally, the ambiguity of species status for reported cryptic taxa may also be a barrier to description, especially if there are also evolving taxonomic practices (e.g., standardizing taxonomic practices against potential “splitting” or “lumping;” such as in cetaceans, Taylor et al., 2017; birds, Cicero et al., 2021).

While taxonomic practices involve the nuances of species criteria (what information qualifies a taxon as a species, e.g., De Queiroz, 1999, 2007), to describe any species is to also assume or imply a particular species concept, which can famously be unclear if not stated or defined by authors (Allmon, 2016; Struck et al., 2017). As Brochu & Sumrall (2020, p. 701) state: “One could kill a large number of systematists by locking them in a room and saying, ‘No one gets out until you all agree on what a species is.’ They are likely to die before they agree.” A species concept determines how criteria (e.g., diagnostic morphology, distinctive life history traits, disjunct geographic distribution, a genetic distance threshold) are interpreted by authors to delineate species. However, directly testing a species' boundary, using whichever chosen species concept—for example, by identifying reproductive incompatibility with species that have naturally occurring populations adjacent or overlapping each other (e.g., at contact or hybrid zones), or via laboratory crosses between closely related species—can be difficult and is rarely done (Chenuil et al., 2019; Knowlton, 1993, 2000; Struck et al., 2017). Instead, morphological features are used to identify most species, assuming differentiating morphology reflects genetic divergence (as a proxy for reproductive isolation). This generalization is not without exceptions, for example, if taxa have few external diagnostic characters (highlighted by Appeltans et al., 2012), or where speciation is known to occur without concurrent morphological change (e.g., corals, Knowlton, 1993, 2000; annelids, Cerca et al., 2019). There are also genetic metrics (e.g., genetic distance, a measure of genetic difference between two species) that serve as proxies for reproductive isolation, but applying these results to delineate species can be taxon‐dependent due to variability in genetic data types and analysis methods (e.g., Carstens et al., 2013; Meyer & Paulay, 2005). Using multiple data types (e.g., genetic, morphological, distribution, paleontological, ecological) as evidence to support a species designation has been recommended (“integrative taxonomy,” e.g., Padial et al., 2010) and used for cryptic taxa but requires significant effort and specialist expertise, and it is unknown how prevalent the integrative approach is (e.g., since a review of articles from 2006 to 2013 using the term, Pante, Schoelinck, & Puillandre, 2015), and studies do not always use this term.

## 3 ECOLOGICAL AND EVOLUTIONARY IMPLICATIONS OF CRYPTIC SPECIES

**FIGURE 1:**
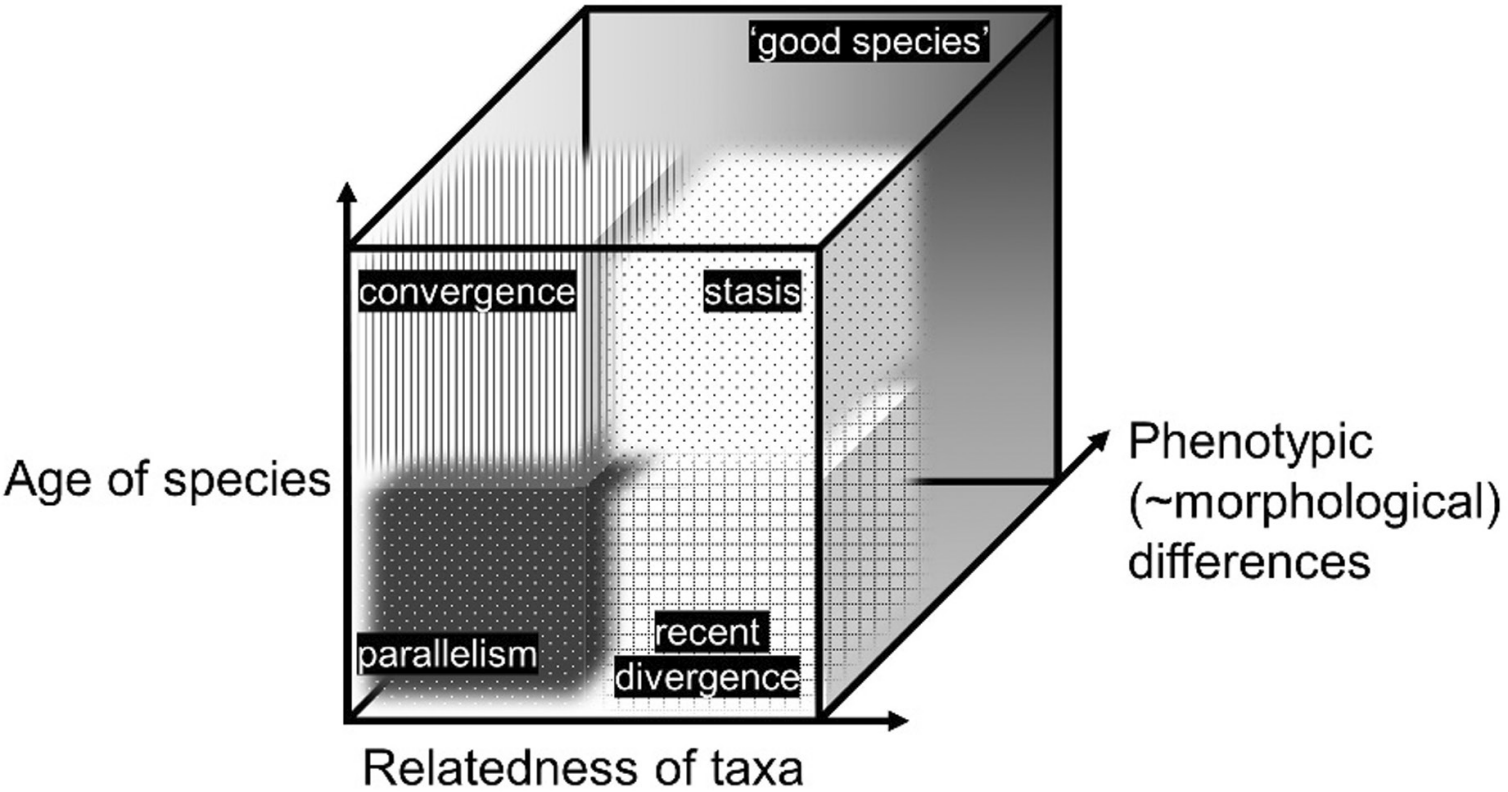
Theoretical space where species may be plotted on three axes: the age of a species, relatedness of taxa, and phenotypic differences. ‘Good species’ are not cryptic and marked by phenotypic differences (Allmon, 2016; Chenuil et al., 2019). Cryptic species occur in the patterned areas of this space, with different possible macroevolutionary causes depending on the estimated species age and relatedness among cryptic taxa (Struck et al., 2017). Blurred boundaries among patterned areas indicate a continuum among attributed speciation causes.

Synthesizing occurrences of reported cryptic species and their attributed speciation processes in a study group can allow for more broadscale discussion of the taxon's evolutionary history (e.g., as discussed in Chenuil et al., 2019; Fišer et al., 2018; Struck et al., 2017; Figure 1).

Within a relatively short timeframe, cryptic species may occur from recent divergence among closely related taxa, or parallel evolution among distant taxa (Struck et al., 2017). These processes may be equivalent to an early phase of speciation, when anticipated morphological distinction has not yet occurred (Chenuil et al., 2019; De Queiroz, 2007; Fišer & Koselj, 2022; Monro, 2022; Struck et al., 2017), sometimes referred to as a “gray zone” on the “speciation continuum” (an approximate scale indicating a range of differentiation from one homogenous species to two distinct species) or the “speciation clock” (the rate at which barriers to gene flow and divergence between taxa accumulate, e.g., Roux et al., 2016). For some groups, divergence may be driven by ecological factors or environments, and be nonvisual cues (among the organisms themselves, e.g., behavior, life history traits, chemical systems) that differentiate morphologically similar, coexisting taxa (“ecological speciation,” e.g., amphipods, Fišer et al., 2018). Recent speciation has been put forward as an important process by which cryptic species arise, but this varies by clade (Bickford et al., 2007; Chenuil et al., 2019; Fišer et al., 2018), and cannot explain all instances of crypticity, as there are morphologically similar lineages that have been separated for millions of years (e.g., coccolithophores, Sáez et al., 2003; amphipods, Fišer et al., 2018; annelids, Cerca et al., 2019).

Over a longer time span, there may be cryptic species from convergent evolution among unrelated taxa that are relatively old (e.g., interstitial fauna, Rundell & Leander, 2010), or from stasis in related species that diverged long ago (e.g., both convergent species and species in stasis are found in jellyfish, Swift et al., 2016; amphipods, Fišer et al., 2018). Stasis is particularly discussed in the context of the fossil record (e.g., Eldredge et al., 2005), in which its demonstration requires statistical comparison against other evolutionary models, principally directional change and unbiased random walk (Hunt, 2007; Hunt et al., 2015; Hunt & Rabosky, 2014). Stasis is also increasingly considered in living species (e.g., Cerca et al., 2019; Struck et al., 2017; Struck & Cerca, 2022), in which its demonstration requires measuring both phenotypic and genetic divergence across a dated phylogeny. If cryptic species result from stasis, then their abundance may have implications for the relative frequency of this evolutionary pattern, and for approximating rates of change in species (e.g., Gingerich, 2019; Hunt, 2007; Hunt et al., 2015).

On a timescale of millions of years, cryptic species pose an especially difficult task, as molecular data are unavailable to assist with identifying morphologically similar species from fossils (“the fossil species recognition problem,” Allmon, 2016, p. 71). Several authors suggest cryptic species in the fossil record are likely numerous (e.g., Hancock et al., 2021; Hoffman & Reif, 1990; Levinton & Simon, 1980; Levinton, 2001, 312 ff.; Pennell et al., 2014), and that this is an argument against being able to study speciation in deep time, especially punctuated equilibrium (a pattern where a species shows relatively rapid morphological change at its divergence, then shows stasis for most of its duration; Eldredge & Gould, 1972). Paleontologists have traditionally responded to such critiques by agreeing they cannot usually—or ever—recognize cryptic species (e.g., MacFadden, 1992, p. 170), but also that the frequency of cryptic species appears to be low in many cases, which would lessen their impact on evolutionary study. For example, by combining data from extant and fossil taxa, some species show that morphological disparity reflects genetic differences (for the general argument, see, e.g., Eldredge, 1989, 108 ff.; Gould, 2002, 785 ff.; for example, see, e.g., Morard et al., 2016 [foraminifera]; Budd et al., 1994; Knowlton et al., 1992 [corals]; Chiba, 2007; Herbert & Portell, 2004; Hills et al., 2012; Michaux, 1987, 1989, 1995 [gastropods]; Jackson & Cheetham, 1990, 1994 [bryozoans]; López‐Carranza & Carlson, 2021 [brachiopods]; Purens, 2016 [crinoids]; Dorit, 1990 [fishes]; Brochu & Sumrall, 2020 [crocodilians]; Pilbrow, 2010 [primates]). If these cases are representative of most taxa, the assumption is made that species in the fossil record (“morphospecies,” or species identified from morphology only) are equivalent to extant biological species (Allmon, 2016; Gould, 2002). If, on the other hand, cryptic species are very common, then the morphospecies of paleontology represent minimal estimates of true biological species and speciation (MacFadden, 1992, p. 173; Allmon, 2016). Resolving the debate on fossil species recognition, therefore, depends on improved knowledge of the actual frequency of cryptic species.

## 4 METHODS

Using the Web of Science Core Collection database, “topics” was searched for the keywords: “cryptic species OR sibling species” AND “marine OR sea OR ocean” AND gastropod*.” This returned 236 results on October 9, 2021, which were then examined by title and abstract for relevance. We did not set restrictions on article publication dates. We excluded conference proceedings and reviews. We selected articles in English that presented original data or analyses on living gastropods with shells as adults. The full text of the resulting 79 articles, published between 1995 to 2021, was reviewed and coded for information by the first author (Appendix S1).

For each reviewed article, metadata (including title, author names, publication year), study purpose, and definitions (of species, and cryptic species) were collected (Appendix S2). All identified gastropod species in the reviewed articles were individually recorded with taxonomic (assigned species name, family, subclass), environmental (sampled habitat, latitudinal zone), and biological (size, juvenile developmental mode) information. For each species reviewed, morphological (shell, whole animal description) and genetic data types (such as mitochondrial, nucleic, allozymes, microsatellites, metabolomic, or total DNA), analysis methods (morphometrics for morphological data; tree, distance, population, marker, and other analyses for genetic data), were also noted (Appendix S3). Different genetic analyses have their own delimitation criteria for taxa (distance‐based methods determine a threshold between genetic variation within and between species, such as using automated barcode gap detection, while tree‐based methods identify transitions between species and population level processes in generated phylogenies; classification after Fišer et al., 2018), or are more directly linked to the study's aims (e.g., identifying variation among populations with population genetics, recognizing a species genetic marker).

We evaluated the cryptic status of studied gastropods (a cryptic species is generally considered by authors to be genetically distinct with no or little morphological differences; in this review, the level of genetic and morphological evidence is used to categorize studied species, after Chenuil et al., 2019) and recorded their suggested macroevolutionary pathways (after Struck et al., 2017; Figure 1), accepting authors' species designations and reasoning as presented. Each named taxon was categorized and considered individually (e.g., if a species was reportedly cryptic with multiple taxa under its name, it was still counted as one). We use three broad categories for identified cryptic species, based on typical kinds of published data available (e.g., genetic analyses, discussion of phenotypic characteristics, indication of spatial distribution): (i) “cryptic species sensu stricto,”  formally named taxa that are genetically identified as species by researchers but *not* morphologically differentiable (for both shell or soft‐body characters, given current data, techniques, expertise), (ii) “cryptic species sensu lato,” which are formally described, genetically *and* morphologically distinct (in shell or internal anatomical features), and (iii) “reported cryptic species,” which are not formally named taxa but those that had a documented genetic difference and could not be confirmed as morphologically cryptic. Based on the authors' data and discussion, “reported cryptic species” were subdivided into (a) species that are probably not cryptic (based on mentioned but unanalyzed morphology), (b) putatively cryptic (based on genetic information), and (c) unconfirmed crypticity (insufficient data). The formal recognition of “cryptic species sensu stricto” and “cryptic species sensu lato” suggests a level of confidence by researchers in assigning species status to cryptic taxa, which differentiates these cases from “reported cryptic species,” where there is much more uncertainty (which could be a result of other reasons, e.g., insufficient sampling of the studied group). Species which did not fall into these groupings were labeled as “not cryptic,” which include taxa that were analyzed but not discussed, and newly described species considered by authors not to be cryptic. Additionally, to gauge the impact of confidently classified cryptic species (cryptic species sensu stricto and sensu lato) on subsequent research, species names were searched in the Web of Science and Zoological Record databases for citations (Appendix S4). While citations can indicate the frequency of a species name use in literature, it may be an underestimate (as reference to the original description of a species name is not required in nontaxonomic works), and we acknowledge citations are only part of the complex and difficult to measure impact of taxonomic efforts.

## 5 RESULTS

**FIGURE 2:**
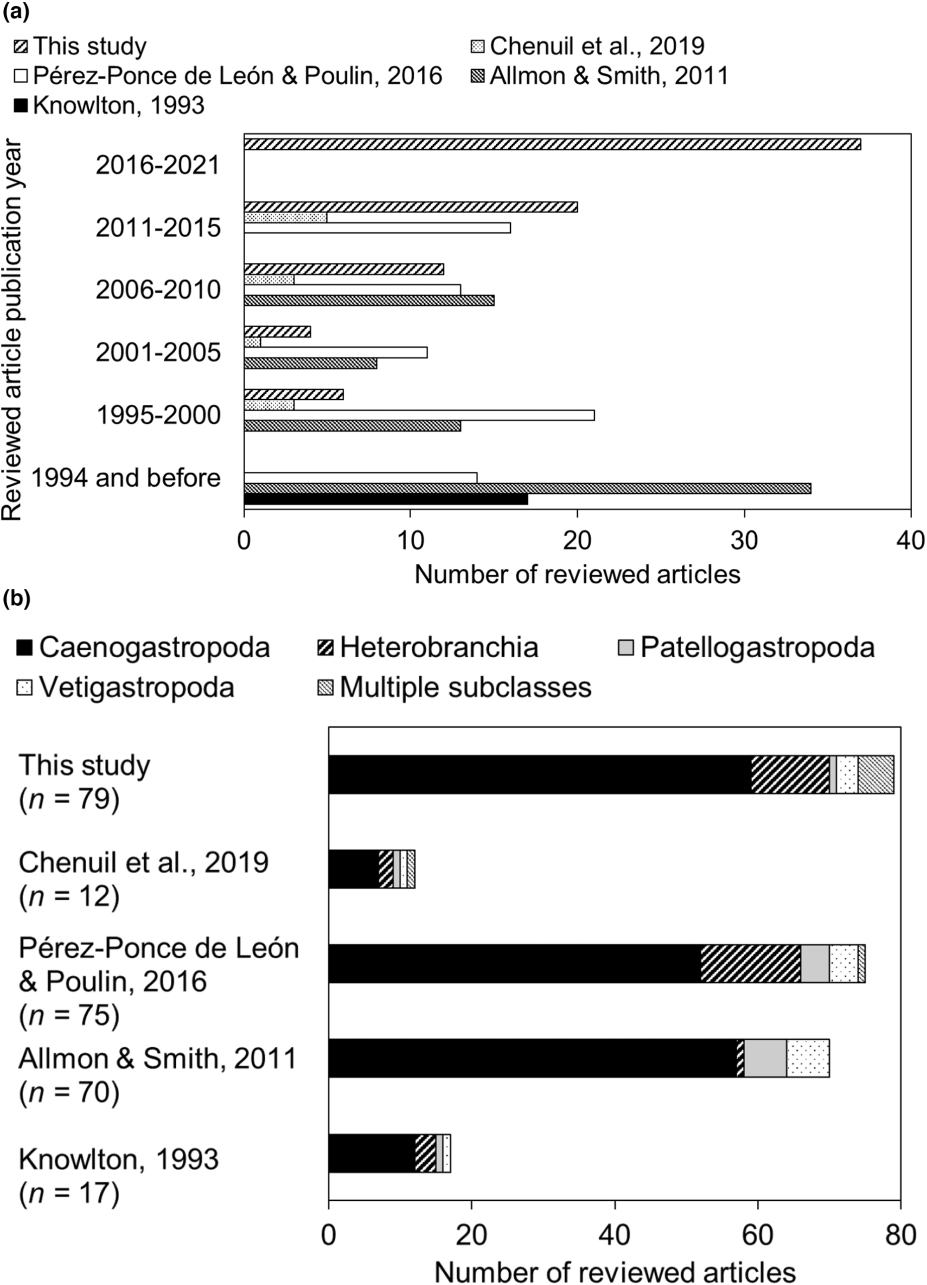
Marine gastropods reviewed in previous literature and this study, as approximated by the number of articles reviewed in respective studies. Note different *x* axis scales. (a) Publication years of articles reviewed by each study. Bars are colored by study. (b) Taxonomic coverage by each study. Total number of reviewed articles per study are indicated in parentheses. Bars are colored by gastropod subclass.

Reviewed articles covered diverse topics, including identifying diagnostic genetic markers, investigating life history aspects, analyzing population structure, examining speciation patterns (e.g., measuring divergence between species, phylogeography), and especially clarifying taxonomy (~50% of articles). Most articles are from the last 5 years (Figure 2a), indicating an increasing rate of cryptic species detection as more molecular techniques, biological groups, and geographic areas are studied. Though including “cryptic species” as a search term has been suggested to disproportionately find publications using the phrase (Li & Wiens, 2022), this does not seem the case in this study. There was similar taxonomic coverage in this study compared with other recent reviews (Figure 2b), and our study also recovered articles that did not find cryptic species (also in Pérez‐Ponce de León & Poulin, 2016). Other reviews on cryptic species (e.g., for multiple phyla, Pfenninger & Schwenk, 2007; with data by gastropod species, e.g., Pérez‐Ponce de León & Poulin, 2016; A. Chenuil, personal communication, May 2022) have either included “cryptic species” in their database searches, or present syntheses without describing their methods (e.g., Allmon & Smith, 2011; Knowlton, 1993, 2000; Table 2). Estimates of cryptic species frequencies are usually presented as a proportion of reviewed articles, with few studies listing results by species.

**TABLE 2:** Comparison of different cryptic species review methods and their estimates. Note that some cryptic species estimates are for the group as a whole, or are more specific (e.g., marine only), and are presented either as a percentage of reviewed articles or number of cryptic species.

Review method	Reference	Number of cryptic species articles or frequency
Mollusca		
Literature search: The Zoological Record, 1978–2006 (58,552 articles)	Pfenninger and Schwenk (2007)	~0.2% of all articles
Literature search (in 2014): Web of Science (402 articles)	Chenuil et al. (2019)	~130 marine cryptic species
Gastropoda		
Literature search: The Zoological Record, 1978–2006 (5407 articles)	Pfenninger and Schwenk (2007)	~0.3% of all articles
Expert opinion	Appeltans et al. (2012)	No data
Literature search: Web of Science, 1978–2015 (121 gastropod articles)	Pérez‐Ponce de León and Poulin (2016)	~60% of articles
Literature search (in 2014): Web of Science (402 articles)	Chenuil et al. (2019) and A. Chenuil (personal communication, May 2022)	17 marine species
Literature search: Web of Science, 1995–2021 (79 articles)	This study	~20%–70% of articles; 135 marine species with shells as adults; ~2%–30% of reviewed species (due to variable confidence in cryptic status)

Authors of articles analyzed in this review often worked in teams to publish multiple articles on potentially cryptic species. Researchers were either experts in a taxonomic group (e.g., Duda et al., 2008, 2009 on Indo‐Pacific species of *Conus*, cone shells), geographic region (e.g., Chinese coast, Yang et al., 2020; Zou & Li, 2016), habitat (e.g., deep‐sea mounts, Castelin et al., 2010, 2012), or generally in cryptic taxa. A few gastropod species were covered by multiple articles, frequently by the same authors. These cases allowed tracking of how research developed on these species over time. For example, a project may include multiple lines of investigation that are better suited to separate publications, as in the Indo‐Pacific species of *Lunella* (turban shells, Turbinidae, genetic analyses; Williams et al., 2011; morphological analyses, Williams et al., 2012). Studying multiple species of interest in the same group (e.g., the Mediterranean reef‐building *Dendropoma* species; worm shells, Vermetidae; Calvo et al., 2009, 2015; López‐Márquez et al., 2018; Templado et al., 2016), or following up an initial cryptic species report with a formal description (e.g., Chilean intertidal slipper shells, Calyptraeidae, Véliz et al., 2003, 2012) illustrates the importance of continued efforts to understand a species.

## 6 DISCUSSION

### 6.1 How cryptic gastropods are studied

The papers analyzed covered 465 marine gastropod species from >110 genera in ~35 families. These species include known species, newly described species (cryptic species sensu stricto and sensu lato), and unnamed species. Most families are represented by <5 articles (~90% of families), which suggests that many gastropods are not well‐known taxonomically (as in past reviews, Figure 2b). Studied taxa were mostly of small or medium body size (up to 50 mm, ~90% of gastropods) and caenogastropods (~75% of taxa). Reviewed species likely reflect sampling accessibility, since few articles explicitly stated they sampled across their taxon's geographic range. Over the reviewed articles' 1995–2021 publication period, most studies were in temperate or tropical latitudes (~75% of taxa), and shallow or coastal habitats (>75% of taxa).

**FIGURE 3:**
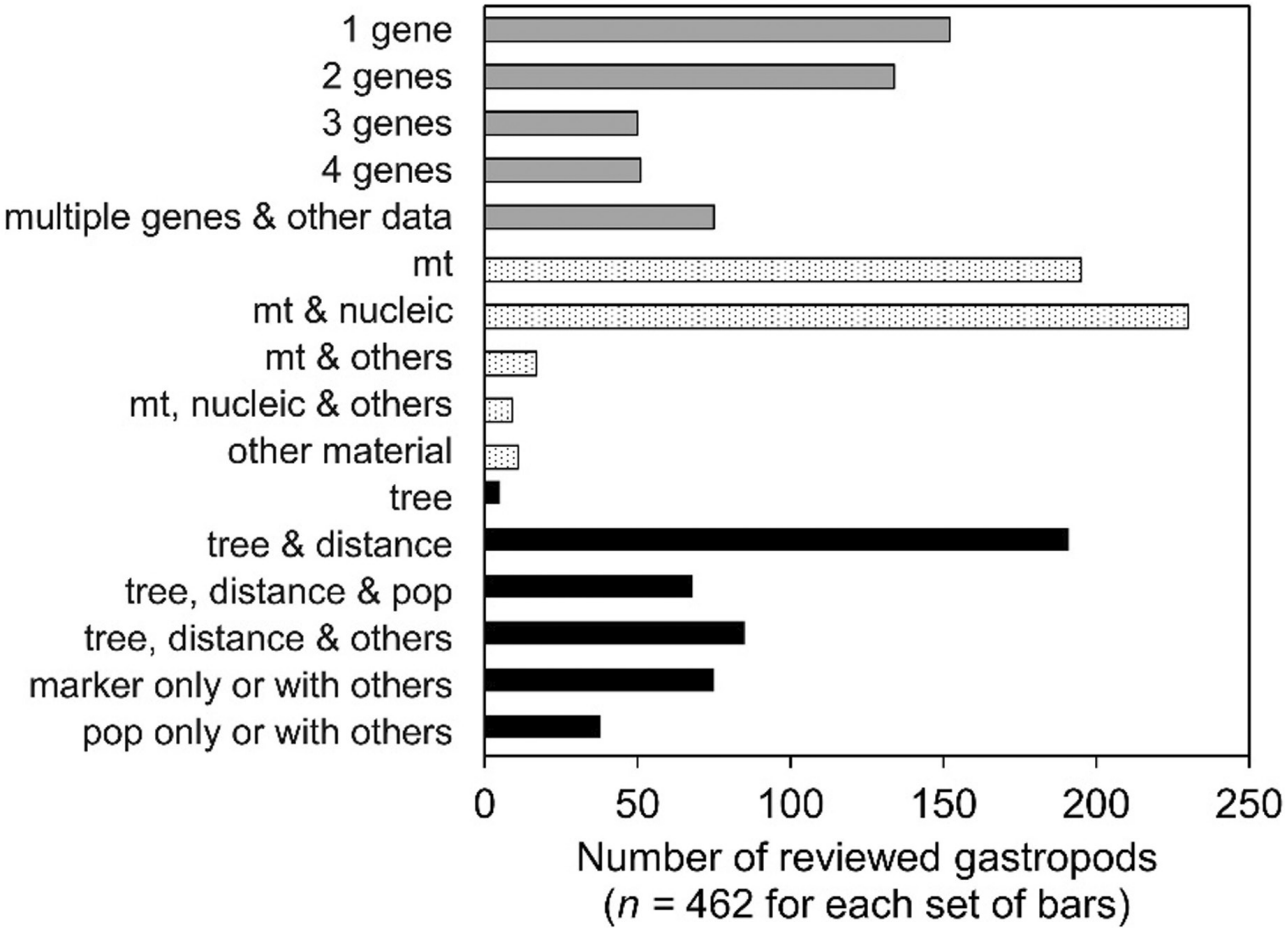
Genetic data and analyses used for reviewed gastropods (*n* = 462 species, three reviewed species did not include molecular data). Each set of colored bars correspond to a different aspect of molecular work conducted and should be considered separately: number of genes used (grey, top), type of material (dotted, middle; ‘mt’ indicates mitochondrial data), and analytical techniques (black, bottom; after Fišer et al., 2018).

The most common method used to identify cryptic gastropods was comparative analyses of species from different genera using mitochondrial DNA in combination with other genetic data (e.g., nuclear genes, microsatellites, allozymes; “mt,” “mt & nucleic,” and “mt, nucleic & others” in Figure 3) or analyses (e.g., distances, tree‐based methods; “tree,” “tree & distance,” “tree, distance & pop,” and “tree, distance & others” in Figure 3), and contextual information (e.g., life history). Overall, there does seem to be a trend in studies over time toward using multiple genes and analyses (as in Taylor & Harris, 2012). For species considered cryptic in this review (*n =* 135), most studies presented mitochondrial DNA and other genetic data, while 30% reported mitochondrial DNA data only. This seems in line with the integrative taxonomy approach, although this term was not normally used. Approximately one‐third of the 465 reviewed species included shell morphology (whether through the authors' own study or citing literature), or both shell and soft part anatomy. Quantitative analyses of shell shape (morphometrics) may not be needed to distinguish species if there are more obvious, discrete phenotypic characters, and they were rarely conducted (~3% of reviewed gastropods). Morphometrics require additional effort and may not fit the scope of articles (e.g., morphometrics were published separately from genetic work for *Buccinum undatum*, common whelk, Magnúsdóttir et al., 2019). Recent gastropod species are described (e.g., cryptic species sensu stricto and sensu lato, Appendix S4) with a combination of molecular data, traditional shell characters, and other available data (e.g., distribution, habitat, life history).

**FIGURE 4:**
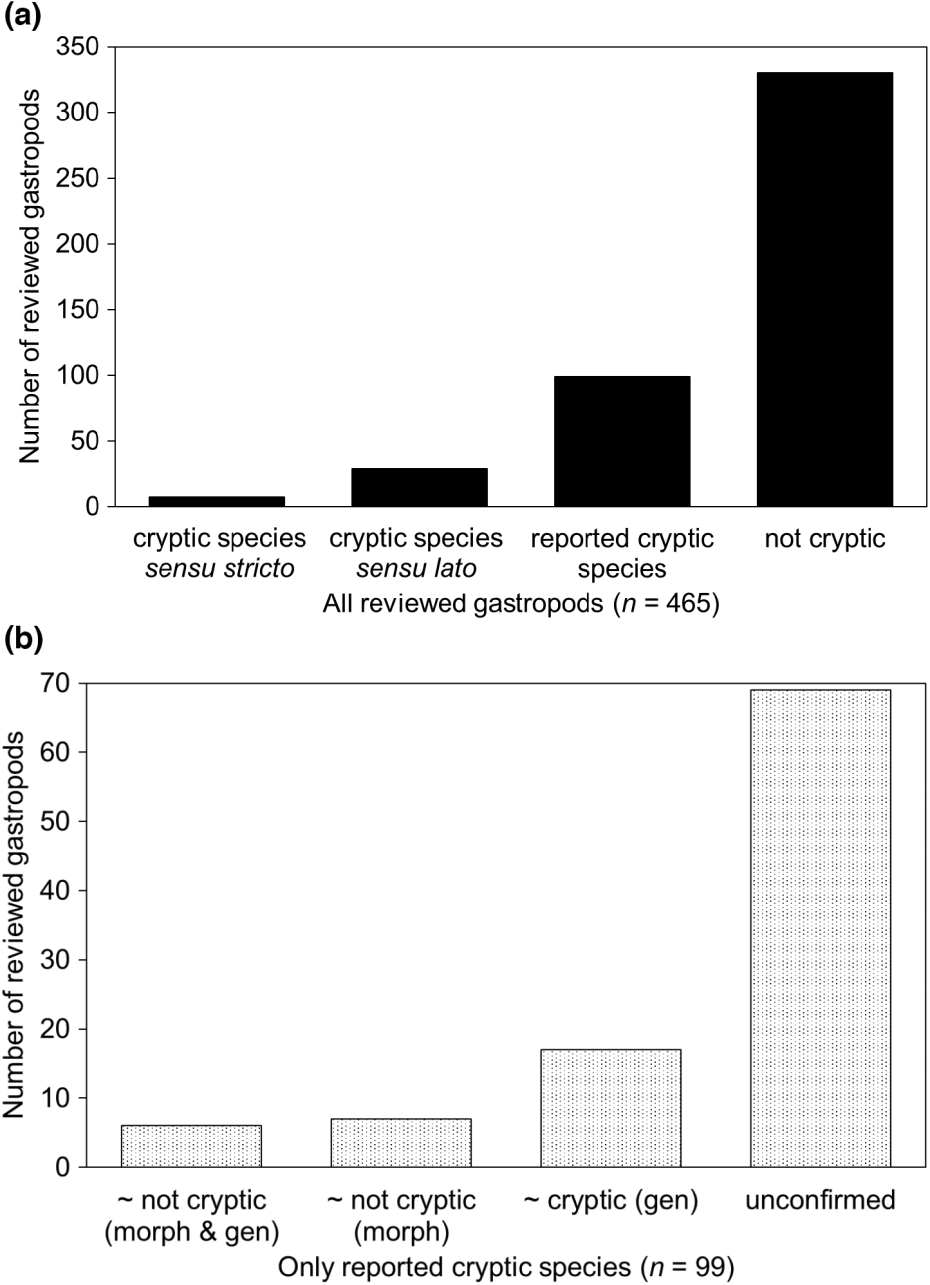
(a) Reviewed shelled marine gastropods (*n* = 465) that are classified as cryptic species sensu stricto, cryptic species sensu lato, reported cryptic species, and not cryptic. (b) Reported cryptic species of gastropods (*n* = 99) are further categorized by evidence available: probably not cryptic, putatively cryptic, and unconfirmed (insufficient data). ‘Morph’ stands for morphological data, and ‘gen’ indicate genetic data. Note y axis scale different between (a) and (b).

**FIGURE 5:**
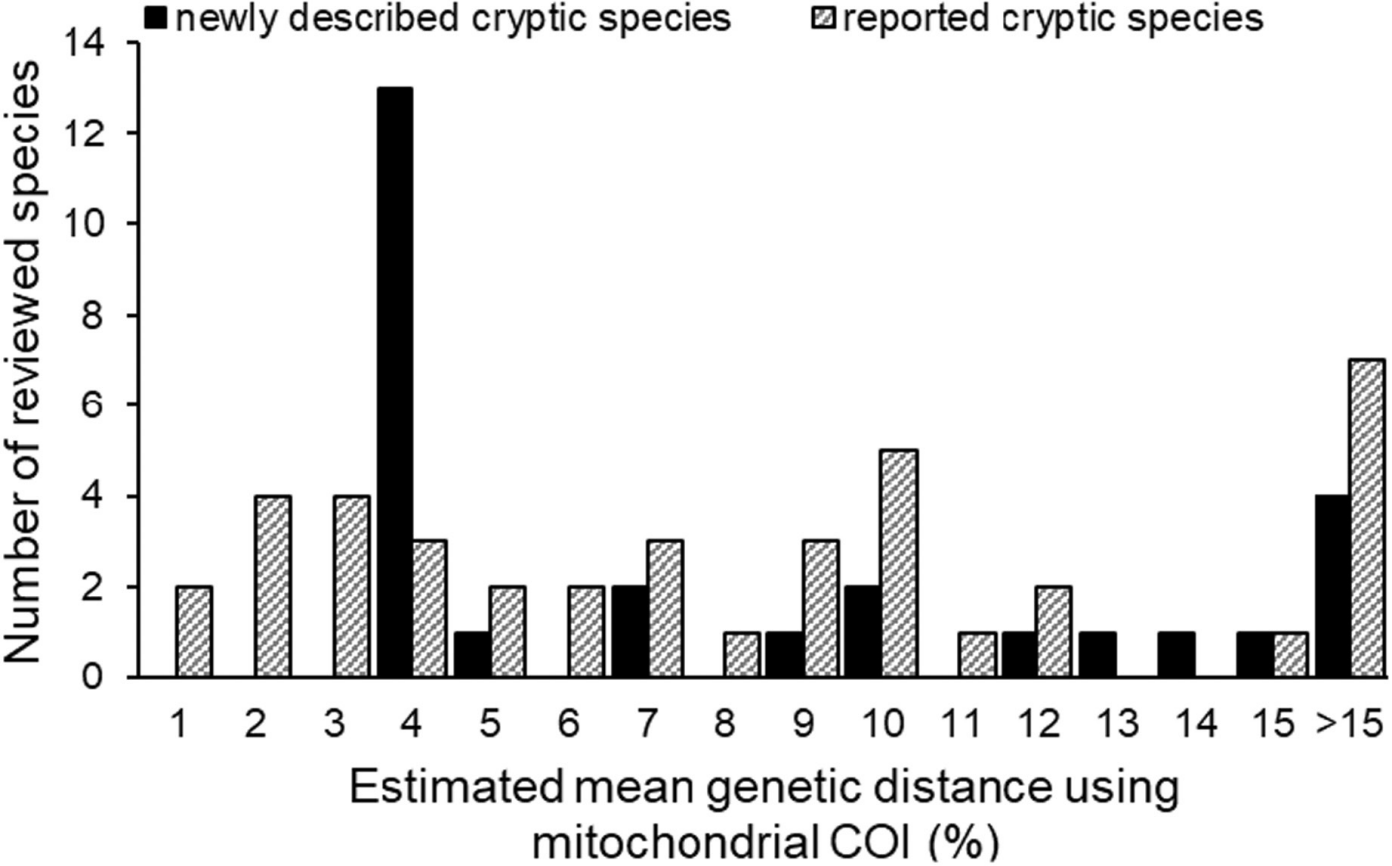
Genetic distances calculated using the Kimura 2‐parameter method with mitochondrial COI from reviewed newly described (cryptic species sensu stricto and sensu lato) and reported cryptic species (*n* = 67, other species used other data, analysis methods, or did not have this information).

**FIGURE 6:**
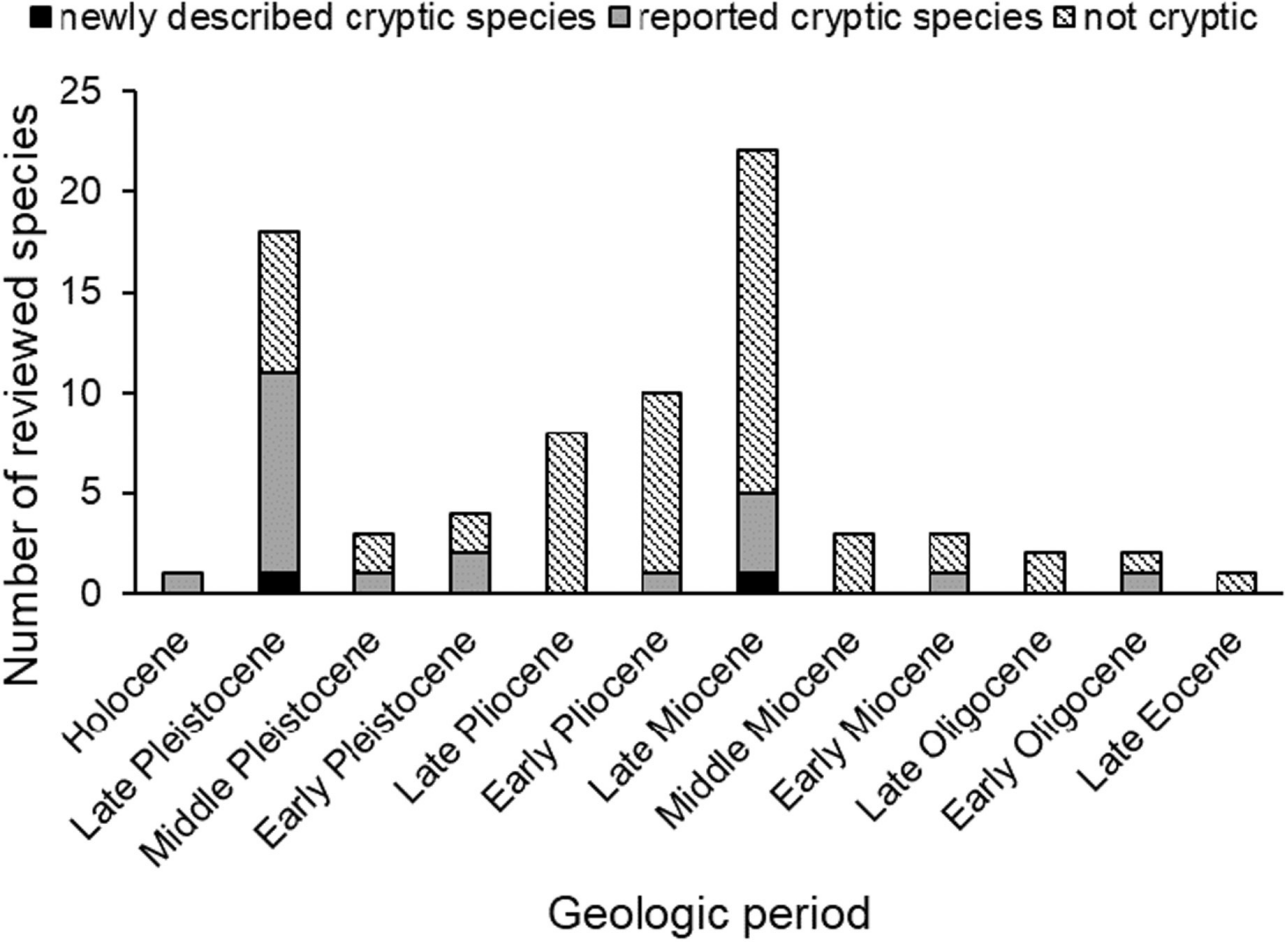
Estimated ages of divergence for reviewed gastropods that had data (*n* = 77; newly described cryptic species are cryptic species sensu stricto and sensu lato). Geologic periods are within the Cenozoic era, listed from present (Holocene, ~12,000 years ago to today, left) to older (Late Eocene, ~38–34 million years ago, right); note periods are unequal in length. For simplicity, species with ages that spanned multiple periods are not shown.

Around 70% of species discussed were confirmed by authors *not* to be cryptic (Figure 4a), meaning they were probably found to have some morphological differences, whether in the external shell, or internal soft parts. However, because these non cryptic species were often not discussed, further classifying how these taxa were identified by researchers (i.e., by shell features only, soft part anatomy, or a combination of both) is not possible. This means that this result on the frequency of morphologically identifiable shelled marine gastropod species cannot be directly applied to their fossil record, but is an upper estimate of recognizable fossil species. Approximately 20% of species were “reported cryptic species” (*n* = 99), which had varying levels of confidence based on the available data presented (the majority of these reports cannot be further categorized in this study, but 13 species are probably not cryptic, and 17 species are putatively cryptic, Figure 4b). There was a considerable range in genetic differences for cryptic gastropods in general (Figure 5). For newly described cryptic species in this review, ~6% were cryptic species sensu lato (taxa with identifiable internal or conchological features, *n* = 29), and ~2% were cryptic species sensu stricto (without diagnostic morphology, *n* = 7, Appendix S4). Few of these named cryptic species were subsequently cited (25%) in articles published during 2010–2021. Cryptic species were rarely associated with suggested speciation mechanisms (12 species; half attributed to stasis, and 3 each to recent divergence and convergence). Studied taxa had a wide range of estimated ages (from Early Oligocene, ~34 million years ago, to Holocene, ~12,000 years ago, Figure 6).

### 6.2 Defining cryptic gastropods

Cryptic species were mostly not defined for reviewed gastropods (~70% of articles, Appendix S2), which made their meaning and application uncertain (as described by Struck et al., 2017). Some articles discussed how species in general are delimited, from which a sense of what authors meant by a cryptic species could be inferred, that is, an undescribed new species with some slight morphological distinction, discovered among phenotypically similar, currently known taxa (~cryptic species sensu lato, see Table 1). There was often insufficient morphological or phylogenetic data to distinguish the various types of cryptic species according to our definitions (Figure 4). Whether authors considered genetically identified clades as “species” is ambiguous because different terms were used synonymously, including “clades,” “lineages,” “evolutionarily significant units,” “molecular taxonomic units,” and “species hypotheses.” Consistent with past reviews (Allmon, 2016; Struck et al., 2017), few authors defined what they meant by “species” or named the species concepts they applied (>75% of articles did not state these, Appendix S2).

Gastropod species were typically delimited by a particular level of genetic divergence and unique morphological characters (in shell and/or soft anatomy), though there was variability in the magnitude of genetic and morphological difference recognized (Figure 5; e.g., for described cryptic species, Appendix S4). Because different delimitation techniques were used among articles (Figure 3), it was unclear which taxa had confirmed genetic isolation, an integral aspect to what constitutes a species and part of proposed methods to study cryptic species (Chenuil et al., 2019; Struck et al., 2017). To comprehensively evaluate a cryptic species report and infer the process by which it arose (Struck et al., 2017), we recommend following up with morphological study and dating of identified species.

### 6.3 Taxonomic treatment of cryptic gastropods

Cryptic gastropods were chiefly discussed in text (“reported cryptic species,” Figure 4, also in Struck et al., 2017), with few formal descriptions (cryptic species sensu stricto and sensu lato, Appendix S4). The lack of consistent cryptic species treatment hinders their inclusion in research beyond their initial discovery but also calls attention to variable methodologies and considerations (e.g., are cryptic species different from typical species?). Though naming cryptic species may inspire further research, and nomenclature codes do not require a morphological diagnosis (e.g., exact genetic sequence differences can be cited, as in Johnson et al., 2015), named cryptic gastropods were rarely cited in papers beyond their initial study (except by authors of the original description or if the species had a particular point of interest, such as distinctive life history). However, limited citations of newly described species (cryptic or otherwise) are only one aspect of a taxonomical publication's impact. The described species in this review are relatively new (around 10 years or less since naming), and some species name use could have been undetected (e.g., if used in an appendix without reference to the original description, as in an ecological survey).

Some authors argue against formally describing cryptic species (e.g., Korshunova et al., 2019), mainly on the grounds that single studies that were not aiming to clarify taxonomy are insufficient in data considered, sampling, and comparative analyses with related taxa to validate species‐level designations. Finding distinguishing morphological characters was implied by many authors to be necessary to delimit species even when genetic analyses suggest potentially new taxa.

### 6.4 Ecology and evolution of cryptic gastropods

In total, about 30% (*n* = 135) of all reviewed species are considered cryptic with varying degrees of confidence (Figure 4), some of which may be due to inconsistent application of the “cryptic species” term, and what evidence is required to substantiate a cryptic species status. A total of 36 species (ca. 8% of those reviewed) were reported as cryptic with higher confidence (7 sensu stricto or 29 sensu lato species). It is difficult to compare these estimates of cryptic gastropods with others, as previous reviews present their results by article, and most do not list their included species (Table 2). Cryptic gastropods were reported in every studied environment and latitude, but the majority were from shallow or coastal habitats (~70% of cryptic species), and tropical or subtropical zones (~60% of cryptic species). Overall, there were similar numbers of cryptic species distributed allopatrically, sympatrically, or both allopatrically and sympatrically with related taxa. Sympatric distributions were often referred to using the term “sympatry,” while cases of species occurring in allopatry did not always use the term “allopatry,” in describing disjunct geographic distribution. The lack of “allopatry” use suggests there may not be enough evidence to explicitly support such a speciation hypothesis (e.g., if the study did not sample across the species' known geographic range), or it is implicitly considered the default speciation mode.

The most common genetic metrics of species divergence were genetic distances using mitochondrial cytochrome *c* oxidase subunit 1 (COI, the gene often used as the basis for DNA “barcoding;” e.g., reviewed by DeSalle & Goldstein, 2019; Taylor & Harris, 2012; marine organisms reviewed in Bucklin et al., 2011; Trivedi et al., 2016) and calculated with the Kimura two‐parameter method (Kimura, 1980). Cryptic marine shelled gastropods had a wide range of interspecific genetic distances (<1% to >15%, Figure 5), which is noteworthy because cryptic species are frequently expected to be closely related to each other and have low genetic distances between species pairs (usually 1–3%; Puillandre et al., 2012). Genetic distances generally did increase with taxonomic level (i.e., generic level differences should be larger than species level comparisons, e.g., Jennings et al., 2010). While most reviewed cryptic species had ≥4% distance with their closest relative, the threshold or “barcode gap” for delimiting species varies by taxon (e.g., Puillandre et al., 2012; Radulovici et al., 2010). Mean COI distances for molluskan sister species pairs have been documented to range from ~6–16%, with a mean of ~11% (Hebert et al., 2003), while the distances among species of the same genus can be greater (e.g., means for two gastropod groups were 17.6% for heteropods, and 21.7% for pteropods, Jennings et al., 2010). However, although barcode gaps have been demonstrated for many sampled mollusks, there are some species where it may not be an appropriate measure (e.g., Layton et al., 2014), especially if the study taxon has not been well‐sampled taxonomically and geographically (e.g., Meyer & Paulay, 2005).

Both conchological and internal features were recommended by authors to substantiate the species status of genetically identified taxa (e.g., Laming et al., 2020; Magnúsdóttir et al., 2019; Malaquias et al., 2016). Cryptic species sensu stricto (*n* = 7) could not be distinguished by morphology. Among cryptic species sensu lato (*n =* 29), most are identifiable with a range of traits (life history for one species, internal anatomy from four species, ~50% from shell only, and ~20% with both internal and external features). Because most cryptic species sensu lato are distinguishable with close examination of conchological attributes (e.g., protoconch, shell sculpture), this gives us confidence in recognizing species in the absence of live material or whole specimens, and species of fossil gastropods. The papers we reviewed show that current taxonomic practices for gastropods continue to emphasize and follow the tradition of using shell features in description (cf., Bieler, 1992).

For the more confidently known cryptic species (sensu stricto and sensu lato, *n =* 36), there were similar numbers of taxa inferred to be planktotrophic (where juveniles feed and develop in the plankton, *n =* 15) and nonplanktotrophic (juveniles feed on egg yolk and spend little to no time in the plankton, *n =* 20, one species did not have an identified developmental mode). The relative frequency of different gastropod developmental modes among described cryptic species suggests that larval mode does not significantly impact whether a gastropod species is cryptic. This would be surprising, because differences in larval ecology have been shown to have broader ecological and evolutionary impact, such as on a species' distribution on a relatively short timescale (with planktotrophic species usually able to disperse over longer distances and have a larger range than nonplanktotrophs, e.g., Jablonski & Lutz, 1983; Krug, 2011), and speciation and extinction on longer timespans (e.g., Jablonski, 1986; Nützel, 2014).

On a macroevolutionary scale, our results do not provide sufficient information (especially on species ages and taxonomic coverage within groups) to clarify the relative frequency and importance of which processes lead to cryptic species. Several microevolutionary‐scale processes were proposed by authors for some species (e.g., allopatric or sympatric speciation, ecological speciation, nonadaptive diversification) but were not included as they are often discussed on a shorter time and at a taxonomically specific scale. Most cryptic species are estimated to have diverged in the Late Pleistocene (~1.8 to 0.012 million years ago, Figure 6), which suggests that cryptic species are more frequent in relatively recent diverged taxa. However, reviewed gastropods all date from the Cenozoic (~66 million years ago to present), indicating taxa generally studied are also fairly (geologically) young.

## 7 CONCLUSIONS

Methodological considerations are inseparable from biological conclusions about cryptic species. From our sample of shelled marine gastropods, >70% of species are morphologically distinguishable (not cryptic), many of which are conchologically distinct and would thus be identifiable for study in the fossil record. Cryptic species are recognized by authors with varying levels of confidence (often depending on available data and techniques), and a nuanced review with explicit definitions and criteria to evaluate these species is necessary to synthesize individual reports for a particular group. Periodic group‐specific reviews would be beneficial in evaluating the frequency of cryptic species, linking their occurrences with prospective ecological or evolutionary causes. A more complete understanding of cryptic species will require the integration of life history, ecological and evolutionary expertise, and fossils of the study taxa, whether as a source of calibrating phylogenies or for discovering potentially unique morphological information to compare with extant species. Fully exploring all these data is an ideal case for an interdisciplinary and comprehensive approach to studying cryptic species, and an effort to do so may advance our current comprehension of the impact of cryptic species on living and extinct biodiversity.

Our results are strongly suggestive but still inconclusive. We, therefore, recommend that future studies on cryptic species:

1. explicitly state or reference a definition of the term “cryptic species” to indicate assumptions and criteria used in categorizing study taxa;
2. cite taxonomic works, including those that mention undescribed or reported cryptic species among the study taxa, so that these occurrences remain relevant and are evaluated after their initial publication;
3. include comments on morphological (or other phenotypic) analyses in genetic‐based studies so it may be clear what data support the species being defined;
4. integrate further with disciplines such as ecology, biogeography, and paleontology when assessing the validity of species assignments, phylogenetic analyses, or ecological conclusions.

## AUTHOR CONTRIBUTIONS

**Caren P. Shin:** Conceptualization (equal); data curation (lead); formal analysis (lead); methodology (lead); visualization (lead); writing – original draft (lead); writing – review and editing (lead). **Warren D. Allmon:** Conceptualization (equal); methodology (supporting); supervision (lead); writing – original draft (supporting); writing – review and editing (supporting).

## CONFLICT OF INTEREST STATEMENT

None.

## Supporting information


Appendix S1–S4

## Data Availability

Datasets are available in appendices.
