# Supplementary material for: How we study cryptic species and their biological implications: A case study from marine shelled gastropods
Source: Ecol Evol. 2023 Sep 5;13(9):e10360. doi: 10.1002/ece3.10360 (PMC10480071; doi:10.1002/ece3.10360)
Supplement: Supplementary file 1 — Appendix S1–S4 [file ECE3-13-e10360-s001.zip › Appendix captions.docx]

Appendix S1. List of information types recorded from reviewed articles.

Appendix S2. List of reviewed articles (n = 79), and their species and cryptic species concepts.

Appendix S3. Information of gastropod species (n = 465) covered by reviewed articles, listed by species.

Appendix S4. Distinguishing morphology and further citations of newly described gastropods (36 species, both cryptic species *sensu stricto* and *sensu lato*) from the reviewed articles.
